# Supplementary material for: Chromothripsis during telomere crisis is independent of NHEJ, and consistent with a replicative origin
Source: Genome Res. 2019 May;29(5):737–49. doi: 10.1101/gr.240705.118 (PMC6499312; doi:10.1101/gr.240705.118)
Supplement: Supplemental Material [file supp_gr.240705.118_Supplemental_file_1.zip › contigs/annotated_contigs/DB55/contig.2.DB55_length_398_mean_cov_3.20603015075.docx]

**DB55_length_398_mean_cov_3.20603015075**

```
CCGATATGCCTTAGAATGGTTTGATTTTTCTTCTAGCCTTTATTGCAGGTTCCCCTGCATCTAGGCTGGAGTGCAGTGGTGCAATCTTG
 >chr19:18582279-18582514 - E=5e-124
GCTCACTGCAACTTCGGCCTCCTGGATTCAAGTAATTCTCCTGCCTAAGCCTCCCAAGTAGCTGAGATTACAGACGCCTGCCACCATGC

CTGGCTAATTTTTGTACTTTTAGTAGAGATAGGGGGTCACCATGTTGGCCAGGCT|GG|CCCCCAAGCCAAGGGTGACCCATCGAACTA
                                                        >chr19:18786483-18786648 - E=8e
TTGGCCGCGGTTCCCACCTATTCCTGCATCCCTGCCCTCTACAGTTCTTCCTTCCAGGTGGAAGACACTTCCCCATTCCAGATGTGGGG
-85
CTCAGCCATGTCATTTGCTTGGCCGAAGACTTATGGGCAGGTCA
```
